# Meiosis Research in Orphan and Non-orphan Tropical Crops

**DOI:** 10.3389/fpls.2019.00074

**Published:** 2019-03-05

**Authors:** Pablo Bolaños-Villegas, Orlando Argüello-Miranda

**Affiliations:** ^1^Laboratory of Molecular and Cell Biology, Fabio Baudrit Agricultural Research Station, University of Costa Rica, Alajuela, Costa Rica; ^2^Department of Cell Biology, The University of Texas Southwestern Medical Center, Dallas, TX, United States

**Keywords:** meiosis, plant breeding, genetic diversity, tropical agriculture, food security, climate change

## Abstract

Plant breeding is directly linked to the development of crops that can effectively adapt to challenging conditions such as soil nutrient depletion, water pollution, drought, and anthropogenic climate change. These conditions are extremely relevant in developing countries already burdened with population growth and unchecked urban expansion, especially in the tropical global southern hemisphere. Engineering new crops thus has potential to enhance food security, prevent hunger, and spur sustainable agricultural growth. A major tool for the improvement of plant varieties in this context could be the manipulation of homologous recombination and genome haploidization during meiosis. The isolation or the design of mutations in key meiotic genes may facilitate DNA recombination and transmission of important genes quickly and efficiently. Genome haploidization through centromeric histone mutants could be an option to create new crosses rapidly. This review covers technical approaches to engineer key meiotic genes in tropical crops as a blueprint for future work and examples of tropical crops in which such strategies could be applied are given.

## Introduction: Crop Yields in a Changing World

Current projections suggest that the world population will increase to 9.6 billion in 2050 and to 10.9–13.2 billion in 2100. Most of this growth may take place in Sub-Saharan Africa, especially Nigeria, followed by Asia (Gerland et al., 2014). Hence, demand for agricultural products is expected to increase by about 50% by 2030 with the increasing global population (Wheeler and Von Braun, 2013), a situation that requires intensifying the food system production (Wheeler and Von Braun, 2013), namely by increasing unit area (yield) (Phalan et al., 2016). This demand is compounded by several problems including (1) insufficient crop yields due to climate change (Zhao et al., 2017) and (2) insufficient crop yield increases with traditional breeding methodologies (Ray et al., 2013).

Furthermore, it is expected that one of the main effects of anthropogenic climate change will be a mean increase of 1.4–5.8°C in the Earth’s surface temperature above the pre-industrial temperature, in the range of 1.4–5.8°C by 2050–2080, caused by the greenhouse gasses carbon dioxide (CO_2_), methane (CH_4_), nitrous oxide (N_2_O), chlorofluorocarbons (CFCs), and ozone (O_3_) (El-Sharkawy, 2014). Modeling suggests that the altered pattern of increase in temperatures can have significant adverse effects on crop yields (Zhao et al., 2017). In maize, each increase in 1°C causes a yield reduction of about 7.4%, and 32% in wheat and rice, respectively (Zhao et al., 2017). Estimates vary for mid-latitude countries, especially South America, which are expected to be not as affected as Eastern Europe, Russia, Northeastern China, and Northwest United States and Canada (Iizumi et al., 2017). Low-income countries at low latitudes may experience the worst effects unless intense technological and management mitigation occurs (Iizumi et al., 2017). Future crop yields are believed to become negative in low-income countries with rapidly growing populations (Ray et al., 2013); in countries such as Guatemala, maize yields are already decreasing (Ray et al., 2013). In this scenario, faster and better improvements on crops will be essential to prevent hunger and sustain the future population of the planet.

## Genomics and Breeding of Orphan Crops

For communities in developing countries, crops such as cassava, sweet potato, yam, plantains, common beans, and millet are of great importance as a food source (Varshney et al., 2012). Most of these crops are not extensively traded, receive little attention in affluent countries, and are grown in marginal environments of Africa, Asia and South America; they are often referred to as “orphan crops” (Varshney et al., 2012). Genomic or bioinformatic resources for orphan crops are usually lacking or underdeveloped (Armstead et al., 2009). Because of lack of access to genotyping, sequencing and computational facilities, scientists have difficulty characterizing these crops (Varshney et al., 2012), and their commonly large, complex and polyploid genomes discourages further research (Kamei et al., 2016).

One viable alternative to study such genomes would be to make use of the available information for model organisms and translate this knowledge to crops (Kamei et al., 2016). Examples of this strategy for applied breeding exist for cassava (Odipio et al., 2017) and for the non-orphan crop cacao (Fister et al., 2018).

Clonal propagation of cassava (*Manihot esculenta*) is thought to have caused a domestication bottleneck, as suggested by the accumulation of deleterious alleles in a heterozygous condition throughout the genome, and may have led to inbreeding depression (Ramu et al., 2017), which is suspected to have reduced yields by 60% (Ramu et al., 2017). The only practical options to purge these mutations is conventional breeding involving sexual reproduction and DNA recombination, perhaps combined with genomic selection and genome editing (Ramu et al., 2017). In cassava (*M. esculenta*), breeding with the wild relative *M. glaziovii* allowed for the transmission of useful traits such as increased water uptake, virus and pest resistance, and apomictic seed development (Nassar and Ortiz, 2010); the latter would be beneficial because it would allow the propagation of hybrids without the need for cuttings that enable viruses and bacteria to contaminate the plants (Nassar and Ortiz, 2010). Introgression of useful traits from wild relatives has also been reported in the breeding of fruit tree papaya (*Carica papaya*) (Coppens d’Eeckenbrugge et al., 2014) and coffee (Herrera et al., 2012) and was proposed in cacao (Dantas and Guerra, 2010). These are tropical cash crops that are not usually considered orphan crops but whose social impact is considerable in developing countries (Myrick et al., 2014; Giuliani et al., 2017; Wickramasuriya and Dunwell, 2018). Therefore, the term orphan crop may not accurately describe scientific and agricultural neglect in all contexts.

## Meiosis and Plant Breeding

Plant breeding of sexually reproducing species relies on the execution of a specialized form of cell division called meiosis (Wang and Copenhaver, 2018). In this process, two rounds of chromosome segregation occur after a single round of DNA synthesis. In the first meiotic nuclear division, maternal and paternal chromosomes, otherwise known as “homologs,” separate, whereas during the second meiotic nuclear division, sister chromatids are segregated (Lambing et al., 2017). The result is the production of recombinant cells, which contain a single copy of the species genome (Lambing et al., 2017; Wang and Copenhaver, 2018).

### Meiotic Recombination

The landmark of meiosis is the process of homologous DNA recombination that occurs during the stage of prophase I, before the first meiotic division. The faithful segregation of homologous chromosomes crucially depends on homologous recombination. This process involves the initial formation of DNA double-strand breaks (DNA DSBs) by the conserved endonuclease SPO11 followed by mechanisms that ensure proper DNA repair (Lambing et al., 2017).

In budding yeast, SPO11 is believed to be cleaved and released by the Mre11–Rad50–Xrs2 (MRX) complex and by Sae2/COM1 (Serra et al., 2018). At the same time endonucleases, such as Mre11 and Exo1 create 3′-overhanging single-stranded DNA that may be thousands of nucleotides in length (Serra et al., 2018). Resected single-stranded DNA is then bound by RAD51 and DMC1 RecA-like proteins, which catalyze the invasion of a homologous chromosome and the formation of a displacement loop (D loop) (Serra et al., 2018). Stabilization of the D loop may occur by template-driven DNA synthesis from the invading 3′end (Serra et al., 2018). Strand invasion intermediates may then progress to second-end capture and formation of a double Holliday junction (dHJ), which can be resolved as a crossover (CO) or non-crossover (NCO) or undergo dissolution (Serra et al., 2018). COs can be further categorized as sensitive (Type I) or insensitive (Type II) to a phenomenon called crossover interference, which prevents closely spaced double COs (Wang and Copenhaver, 2018). The formation of Type I COs is believed to be regulated by the MSH4/MSH5 MutS-related heterodimer, MER3 DNA helicase, SHORTAGE OF CROSSOVERS1 (SHOC1) XPF nuclease, PARTING DANCERS (PTD), ZIP4/SPO22, HEI10 E3 ligase, and MLH1/MLH3 MutL-related heterodimer (Serra et al., 2018). Within this pathway, the HEI10 E3 ligase gene shows dosage sensitivity, which means that copies increase crossovers throughout euchromatin. Type II COs form by a different MUS81-dependent pathway, account for about 15% of crossovers and do not show interference (Serra et al., 2018). NCOs are thought to be generated by an alternate pathway called synthesis-dependent strand annealing (SDSA). SDSA follows the same initial steps as DNA DSB repair until second-end capture, when the invading strand instead dissociates, and the newly synthesized 3′ DNA anneals to the single-strand 3′end on the opposite side of the original break. Gap-filling DNA synthesis and ligation result in an NCO (Wang and Copenhaver, 2018).

The production of viable offspring and the generation of new combinations of traits/alleles in plants crucially depends on the balance of COs/NCOs; for instance, if formation of an obligate CO is absent, there is non-disjunction, whereas the opposite situation of elevated CO level does not lead to inviability (Crismani et al., 2012). Plant breeding also relies on the formation of meiotic crossovers to combine favorable alleles into elite varieties (Mieulet et al., 2018). However, meiotic crossovers are rare, normally 1–3 per chromosome, which limits the efficiency of the breeding process and genetic mapping (Mieulet et al., 2018). Therefore, the manipulation of meiotic recombination to increase the CO/NCO ratio is of capital importance to improve the ability of plant breeders to obtain better combinations of traits and faster.

For instance, *Arabidopsis* has approximately 150–250 DSBs per meiosis, as estimated by immunostaining of DSB markers, such as γH2A.X, RAD51, and DMC1. However, the repair of these DSBs results in the formation of only about 10 COs, which suggests the activity of inhibitory mechanisms, called anticrossover factors, that prevent CO resolution (Wang and Copenhaver, 2018). NCO repair of strand invasion events is believed to be promoted by multiple, non-redundant pathways that may include the proteins FANCONI ANEMIA COMPLEMENTATION GROUP M (FANCM), MHF1, MHF2, FIDGETIN-LIKE1 (FIGL1), RECQ4A, RECQ4B, TOPOISOMERASE3α (TOP3α), and MSH2 (Mercier et al., 2015). The action of these NCO pathways results in repair of about 90% of all initial meiotic DNA DSBs as NCOs (Mercier et al., 2015). As stated earlier, the formation of crossovers is also regulated by activity of the *HEI10* meiotic E3 ligase gene (Ziolkowski et al., 2017), and additional copies of the gene enhance the effectivity of the process (Ziolkowski et al., 2017), especially in the *recq4a/recq4b* mutant background (Serra et al., 2018). An R264G polymorphism in the C-termi-nus of HEI10 is also believed to enhance recombination by promoting protein function or expression timing (Ziolkowski et al., 2017).

Analysis of tomato cv. Micro Tom EMS-mutant lines for the antihelicase RECQ4 indicated a 2.7-fold increase in recombination, and a similar outcome was reported for rice Dongjin/Nipponbare F_1_ hybrids, which are *recq4/fancm* mutants; therefore manipulation of the crossover formation in crops is feasible (Mieulet et al., 2018).

### Genome Haploidization Using Modified Centromeric Histones/Apomixis

In the model plant *Arabidopsis thaliana*, haploid clonal plants can be obtained from seeds by altering the coding sequence for the centromere-specific histone CENH3 (CENP-A in humans), which is universal in plant species (Ravi and Chan, 2010). On crossing *cenh3* homozygous mutants expressing an altered *CENH3* sequence with the wild-type, chromosomes from the mutant are eliminated in the zygote, which results in haploid progeny. Haploids are then spontaneously converted into fertile diploids via meiotic non-reduction, which allows for propagation of the genotype of choice (Ravi and Chan, 2010). Changes in the naturally hypervariable N-terminal tail of CENH3 cause segregation errors and chromosome elimination (Maheshwari et al., 2015). Comparison of CENH3 protein sequences from more than 50 plant species showed that the N-terminal tail region is highly variable, whereas the C-terminal histone fold domain (HFD) is relatively conserved across species. A key HFD mutation (P82S) caused by a single nucleotide substitution induced haploidy in *Arabidopsis* (Kuppu et al., 2015). This mutation occurs in crops such as cassava, papaya, bananas, soy, maize, and rice and may be exploited for plant breeding purposes (Kuppu et al., 2015). A similar mutation (L130F) causes inactivation of centromere loading in barley (Karimi-Ashtiyani et al., 2015). It has been suggested to combine this approach with the simultaneous inactivation of meiotic genes *OSD1* (*OMISSION OF SECOND DIVISION*, a negative regulator of the *Arabidopsis* APC/C during meiosis), *REC8* (required for proper separation of sister chromatids during meiosis I) and *SPO11*. The inactivation of these three genes leads to the *MiMe* genotype: *Mitosis instead of Meiosis*, and it is believed that a combination with *CENH3* engineering may produce asexual seeds (Ishii et al., 2016). Alternatively, the *MiMe* genotype may be combined with ectopic expression in the egg cell of the *BABY BOOM 1* (*BBM1*) sperm transcription factor to induce parthenogenesis and asexual seed development (apomixis), as shown in rice cultivar Kitaake (*Oryza sativa* L. ssp. *japonica*) (Khanday et al., 2018).

## Tropical Crops Amenable for Meiotic Gene Manipulation

### Cassava (*Manihot esculenta* Crantz)

**FIGURE 1:**
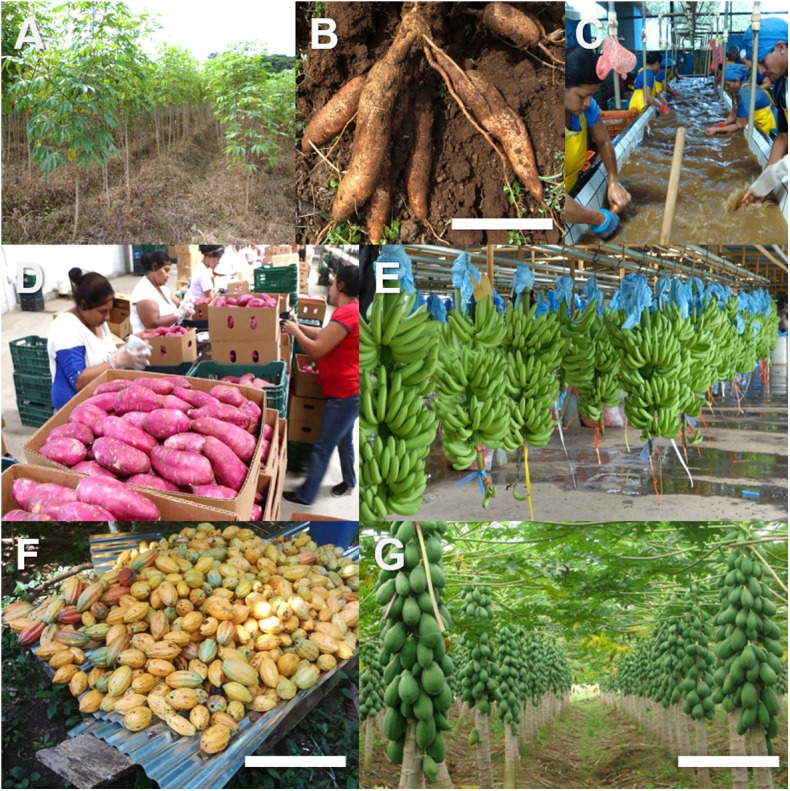
Production of tropical crops in Central America. **(A)** Cassava farm in Northern Costa Rica. **(B)** Cassava tubers being harvested (Costa Rica). **(C)** Cassava processing plant (Costa Rica). **(D)** Sweet potato packaging facility in Honduras. **(E)** Banana processing facility in Costa Rica. **(F)** Papaya farm in Costa Rica. **(G)** Harvest of cacao in Costa Rica. Scale bars: **(B,G)** 25 cm, **(F)** 50 cm. Image credits: **(A)** Alfredo Durán (University of Costa Rica), **(B,C)** Helga Blanco-Metzler (University of Costa Rica), **(D)** La Prensa Newspaper (Honduras), **(E)** Rodríguez Chaparro and Héctor Osvaldo, National Distance University (UNED) image repository, Costa Rica, **(F)** Eric Mora-Newcomer (University of Costa Rica), **(G)** Óscar Brenes, Foundation for Agricultural Research (FITTACORI, Costa Rica).

The cassava genome is 742 Mb in size (2*n* = 36) and to contain 34,483–38,845 functional genes (Wang et al., 2014). Cassava is the main source of starch for 700 million people around the world (Wang et al., 2014; Figure 1A–C). Surprisingly, up to 19% of all coding single nucleotide polymorphisms are believed to be deleterious (Ramu et al., 2017), which may explain its poor root yield of only 13.6 tons per hectare (Wang et al., 2014). Meiosis in interspecific hybrids between *Manihot esculenta Crantz* and *Manihot neusana Nassar* lead to the formation of restitution nuclei and micronuclei (Nassar et al., 1995), caused by defects during anaphase I (Nassar et al., 1995). Backcross generations 1–4 were aneuploid and eventually sterile (Nassar et al., 1995). Light microscopy analysis of embryo sacs in *M. neusana* suggested that 1.5% of all ovules were apomictic, and F_2_ hybrids between *Manihot esculenta* and *M. neusana* appeared to be fully apomictic (Nassar et al., 2000). Gene editing with the CRISPR/Cas9 system in calli of cassava is possible (Odipio et al., 2017), which suggests possible editing of key meiotic genes, especially those related to CO/NCO formation. One possibility in cassava would be to facilitate outcrossing by targeting homologs for *FANCM*, *FIGL1*, *RECQ4A*, and *RECQ4B* as done in tomato and rice (Mieulet et al., 2018). Formation of double haploids by Targeting Induced Local Lesions in Genomes (TILLING) and engineering of *cenh3* mutants has also been suggested for cassava (Kuppu et al., 2015).

### Sweet Potato (*Ipomoea batatas* Linn.)

Sweet potato is one of the oldest domesticated crops in the Americas (Kyndt et al., 2015), and is the only cultivated species among the 15 in the section batatas of the family Convolvulaceae (Becerra Lopez-Lavalle, 2002; Figure 1D). Cultivated sweet potato is autoallohexaploid (2*n* = 90), although wild tetraploid specimens (2*n* = 60) have been reported (Becerra Lopez-Lavalle, 2002; Kyndt et al., 2015). Most of the world’s production is concentrated in China (Wang et al., 2010). The large genome (2205 Mb) contains approximately 56,516 unigenes; 35,051 have been identified (Wang et al., 2010). Sweet potato is one of the most efficient crops in terms of dry-matter productivity and is a model for carbohydrate storage and tuber formation (Wang et al., 2010). Unfortunately, sweet potato is vegetatively propagated, and it is prone to accumulate and disseminate geminiviruses (Paprotka et al., 2010). 2*n* pollen and polyads are formed in tetraploid accessions, so conventional breeding is difficult (Becerra Lopez-Lavalle, 2002). However, work in tetraploid *Arabidopsis arenosa* suggests that meiotic chromosome segregation may be improved by bringing chiasmata number down to one per bivalent because limiting crossovers to one per chromosome prevents multivalent associations (Yant et al., 2013). Results from TILLING and cytological analyses suggest that in polyploid accessions, homoeologous recombination may be reduced by selection of putative specific amino acid sequences in genes involved in sister chromatid cohesion, axis formation, synapsis and recombination, namely *ASY1*, *ASY3*, *SYN1/REC8*, *SMC1*, *PDS5*, *ZYP1a*, and ZYP1b (Wright et al., 2015). In some cases, these changes may reduce DNA binding, and in some cases, they may reduce phosphorylation of the putative protein (Wright et al., 2015). The most notable changes were K40E in the DNA-binding HORMA domain of ASY1; T265I and L268V in ASY3; S242F and S527Y in PDS5; and F595S and Q923K in SMC1 (Wright et al., 2015). Selection of such residues in sweet potato accessions combined with genome editing might help improve meiotic chromosome segregation, viability and facilitate conventional breeding. Alternatively, decreased ASY1 activity in wheat transgenic lines promotes homoeologous pairing (Yant et al., 2013), so overexpression of ASY1 might be useful to reduce CO formation.

### Banana (*Musa* sp.)

Banana and plantain are major staple foods and are a source of income for millions in tropical and subtropical regions (Tripathi et al., 2013; Figure 1E). Most bananas and plantains grown worldwide are produced by small-scale farmers for home consumption or for sale in local and regional markets. Many pests and diseases significantly affect *Musa* cultivation (Tripathi et al., 2013).

The genome of *M. acuminata* (2*n* = 22) is 523 Mb in size (D’hont et al., 2012). Cultivated bananas are mainly triploids, and breeding mostly involves crossing fertile triploids with diploids to obtain tetraploids, which are then crossed to diploid accessions to obtain triploid cultivars (Muiruri et al., 2017). Modification of *Arabidopsis* CENH3 by replacing the N-terminal tail with that of the variant H3.3 and tagging it with GFP resulted in haploid formation (Muiruri et al., 2017), an outcome that if properly exploited would be useful to breed new bananas (Muiruri et al., 2017). The rationale is as follows, work in barley interspecific hybrids has shown that CENH3 is required for kinetochore function (Sanei et al., 2011); if the CENH3 sequences from both parents are very divergent during early embryogenesis, centromere activity will remain in both parental genomes (Sanei et al., 2011). However, chromosomes of the male will start to lag because of centromere inactivity during anaphase, subsequently forming micronuclei. Finally, micronucleated male chromatin will degrade, and a haploid maternal embryo will develop (Sanei et al., 2011).

Hypothetically, genotyping banana accessions for divergent *CENH3* alleles could be used to produce natural triploid hybrids (Muiruri et al., 2017). *CENH3* sequences were analyzed in the accessions “Calcutta 4” and “Zebrina GF” from *M. acuminata*, in *M. balbisiana*, and in the commercial interspecific triploids “Sukali Ndiizi,” “Pisang Awak” and “Gros Michel” (Muiruri et al., 2017). The genotype “Calcutta 4” and “*M. balbisiana*” have one each, “Gros Michel” and “Pisang Awak” has two, “Zebrina GF” has four and “Sukali Ndiizi” have seven (Muiruri et al., 2017). These sequences are highly variable in the N-terminal tail and show specific P-to-A and G-to-E amino acid substitutions within the HFD that may be used to determine crosses (Muiruri et al., 2017).

### Cacao (*Theobroma cacao* Linn.)

Cultivation of *T. cacao*, the tropical tree that produces cocoa beans, is a key export activity for many developing countries, especially from Africa (Fister et al., 2018; Figure 1F). Thus, a reliable and sustainable output is important to guarantee the livelihoods of 6 million small-scale cacao farmers around the world (Fister et al., 2018; Wickramasuriya and Dunwell, 2018). Cacao seeds are a rich source of polyphenolic antioxidants that may prevent cancer or delay/slow the progression of cancer and serve as cardioprotective agents (Wickramasuriya and Dunwell, 2018).

The two most serious diseases of cacao are caused by the fungi *Crinipellis perniciosa* (witch’s broom disease) and *Moniliophthora roreri* (frosty pod rot) (Aime and Phillips-Mora, 2005). Annual losses are 30% (Argout et al., 2011).

*T. cacao* L. is a diploid tree species (2*n* = 20) from the Malvaceae family that is endemic to South American rainforests. It is believed to have been domesticated approximately 3,000 years ago in Central America (Argout et al., 2011). The genome of the Belizean Criollo genotype B97-61/B2 is 430 Mb in size and is rich in retrotransposons (Argout et al., 2011). Cacao displays a late-acting self-incompatibility syndrome, which results in failure of karyogamy after discharge of sperm cells into the embryo sacs (Gibbs, 2014). Selfed pistils in this species abscise 3 days after post-pollination (Gibbs, 2014). Five self-incompatibility genes are proposed to regulate the process, showing dominance and equal effects (in both pollen and pistil) with the sequence of importance S_1_ > S_2_ = S_3_ > S_4_ > S_5_ (Gibbs, 2014); however, no genes have been characterized molecularly. Meiosis has been analyzed in the diploid clones T85/799 (derived from a cross between two Upper Amazon varieties), T28 (a Venezuelan Criollo) and TF6 (from Ghana) (Martinson, 1975). Chromosome segregation at anaphase is regular and laggards are rare. Chiasma frequency was estimated at 9.00–9.35 per cell (Martinson, 1975). These results suggest that the chiasma frequency is less than the basic chromosome number, which implies that during meiosis, univalents are present in most cases and hint at defects in Type I CO formation. Unfortunately, no recent descriptions of cacao meiosis are available.

Development of double haploids is difficult in cacao and has even involved irradiation of pollen at 50 and 100 Gy to induce inhibition of the division of the generative nucleus. The only way to obtain haploid plantlets may be *in vitro* ovary culture (Falque et al., 1992). Cacao is amenable to transformation with CRISPR/Cas9 by using detached leaves (Fister et al., 2018), and haploid clonal plant formation might be possible, as was suggested for banana (Muiruri et al., 2017). Alternatively, deregulation of anticrossover activity by the cacao homologs of *FANCM* and *RECQ4* may facilitate the introgression of wild traits in cacao elite cultivars. Comparison of sequencing results across several accessions in the West Indies and Costa Rica including Criollo, Amelonado and Nacional cultivars suggests that during the process of domestication, there may have been a strong selection for genes involved in the metabolism of protecting anthocyanins and the stimulant theobromine, coupled with a general decrease in population fitness and reproductive success (Cornejo et al., 2018).

### Papaya (*Carica papaya* Linn.)

Papaya is a fruit tree cultivated in tropical and subtropical regions and is known for its nutritional benefits and medicinal applications (Ming et al., 2008; Figure 1G). Papaya is not considered an orphan crop, but its consumption has a considerable impact on the health and well-being of vulnerable populations. Indeed, consumption of its fruit may prevent vitamin A deficiency, a cause of childhood blindness in tropical and subtropical developing countries (Liao et al., 2017). The largest producers of papaya are Brazil, Indonesia, Ethiopia, Congo, Thailand, Guatemala, and Colombia (Fuentes and Santamaría, 2014).

Papaya belongs to the small family Caricaceae, which contains 6 genera and 35 species (Liao et al., 2017). It is a diploid (2*n* = 18) with a small genome of 372 Mb and possesses a primitive sex-chromosome system (Ming et al., 2008). In papaya, females are XX while maleness and hermaphroditism are controlled by slightly different sex-specific Y chromosome regions: Y^h^ (HSY) in hermaphrodites and Y (MSY) in males (VanBuren et al., 2015). Hermaphrodite flowers give rise to oblong fruits that are commercially desirable (Jiménez et al., 2014). Both HSY and MSY loci are 8.1 Mb long and are located on chromosome 1, the largest. Recombination with the X chromosome is suppressed, and any combination of the Y and Y^h^ loci (YY, YY^h^, or Y^h^Y^h^) is inviable (VanBuren et al., 2015).

Hybridization of papaya with the wild relative *Vasconcellea quercifolia* allowed for successful introgression of resistance to Papaya ringspot virus into backcross generations 3 and 4, as determined by serological tests and field evaluation in infested plots (Siar et al., 2011). The process is laborious and requires *in vitro* culture of embryos (Siar et al., 2011). Nonetheless, interspecific hybridization was found an important tool for breeding new papaya cultivars (Siar et al., 2011). In wheat, the *ph1b* deletion line has been exploited in crosses with wild relatives to allow for exchange between chromosomes at meiosis (Rey et al., 2018). The *ph1b* deletion has been shown to correspond to the *ZIP4-B2* gene, a factor that regulates the formation of type I COs (Rey et al., 2018). When this mutation is combined with a nutrition regime rich in Mg^2+^, the mean number of COs increases to 12 per cell as compared with 1–7 in *ph1b* mutants and 1 in the wild type (Rey et al., 2018). High phosphate has also a positive effect in barley meiosis, and was shown to increase chiasmata formation from 7,7 per cell in the control to 10,6 (Fuchs et al., 2018). Therefore, gene editing in papaya combined with enhanced nutrition with Mg^2+^ or phosphate might facilitate outcrossing with wild relatives.

## Conclusion

Tropical crops, considered orphan or not, are the key to preventing hunger, guaranteeing good health and creating economic growth in developing countries (Kamei et al., 2016; Liao et al., 2017). The translation of current knowledge of meiotic processes such as homologous recombination, and CO formation may help produce new varieties that are enriched in desirable wild traits. The application of one particular approach to manipulate meiosis in these crops may depend on factors that go beyond the scope of this review and may vary from what is suggested. However, by first outlining the possibilities, we hope to encourage research into the regulation of meiotic processes in tropical crops and applied translational work.

## Author Contributions

All authors listed have made a substantial, direct and intellectual contribution to the work, and approved it for publication.

## Conflict of Interest Statement

The authors declare that the research was conducted in the absence of any commercial or financial relationships that could be construed as a potential conflict of interest.
